# Do Lasers Have an Adjunctive Role in Initial Non-Surgical Periodontal Therapy? A Systematic Review

**DOI:** 10.3390/dj8030093

**Published:** 2020-08-16

**Authors:** Donald Coluzzi, Eugenia Anagnostaki, Valina Mylona, Steven Parker, Edward Lynch

**Affiliations:** 1 School of Dentistry, University of California, San Francisco, CA 94143, USA; 2 Leicester School of Pharmacy, De Montfort University, Leicester LE1 9BH, UK; eugenia.anagnostakis@my365.dmu.ac.uk (E.A.); vasiliki.mylona@my365.dmu.ac.uk (V.M.); steven.parker@my365.dmu.ac.uk (S.P.); edward.lynch@hotmail.com (E.L.); 3 School of Dental Medicine, University of Nevada Las Vegas, Las Vegas, NV 89106, USA

**Keywords:** adjunctive, dentistry, laser, periodontal, periodontitis, randomized clinical trials, therapy

## Abstract

(1) Background: dental lasers have numerous applications for periodontal therapy which include surgical procedures of soft tissue and osseous structures, and non-surgical treatments such as pathogen reduction, removal of surface accretions, and photobiomodulation. The aim of this review was to evaluate the scientific literature to ascertain whether lasers have a beneficial role when used adjunctively in initial non-surgical periodontal therapy. (2) Methods: A PubMed search was performed specifically for randomized clinical trials where a dental laser was used adjunctively for initial periodontal therapy on human patients published from January 2010–April 2020. The first search identified 1294 eligible studies. After additional criteria and filters were applied, 20 manuscripts were included in this review. (3) Results: The chosen manuscripts reported on investigations into initial therapy for patients diagnosed with chronic periodontitis. After periodontal charting, conventional instrumentation such as hand and ultrasonic scaling was performed on all patients in the studies, and then a test group or groups of patients were treated adjunctively with a laser. That adjunctive laser group’s periodontal findings showed various degrees of improved health compared to the group treated with only conventional methods. (4) Conclusion: This systematic review found that 70% of the included studies reported significantly better outcomes in certain clinical parameters, but no improvement in others. The remaining 30% of the manuscripts reported no significant difference in any of the measurements. With consideration to correct parametry, lasers have an adjunctive role in initial non-surgical periodontal therapy.

## 1. Introduction

Chronic periodontal disease continues to be a significant health problem that commonly occurs in adults [1]. Certain risk factors including genetics, medical history, and lifestyle also exist that can increase the disease’s severity and those factors continue to be discovered [2].

This disease is a multi-factorial, predominately chronic inflammatory process whereby microbial deposits associated with the gingival tissues produce toxins that evoke tissue reaction, loss of bone and gingival support; untreated, it can lead to tooth mobility, migration and loss [3]. Periodontitis is a slowly progressing oral infection of the soft and hard tissues surrounding the teeth. The primary etiology is the presence of pathogens in a biofilm which, when left intact on the gingiva or tooth surface, can lead to soft tissue inflammation. Subsequently, increasing pathogen population can then invade the connective tissue attachment while causing apical migration of the epithelial attachment apparatus with resulting alveolar bone resorption and tooth loss. Treatment always begins with initial periodontal therapy whose purpose is to remove the aforementioned biofilm and calcified deposits, known as calculus. This initial therapy will continue with periodic evaluations to assess the result and is sometimes termed ‘Phase 1 therapy’ which implies that subsequent surgery (Phase 2) would have to follow if the periodontal infection cannot be reversed [4]. The widely used protocol is known as periodontal debridement [5] and can result in improvement of clinical indices such as decreased probing depths and reduction of inflammation. This non-surgical approach continues to be the hallmark of periodontal therapy [6]. However, it has been known for some time that the debridement is usually incomplete because of the difficult access to some areas of the periodontium [7]; moreover, one study has shown that sonic powered scalers could have little effect on pathogen reduction [8]. The use of systemic or locally delivered anti-infective medications has been utilized to supplement debridement with benefits and limitations [9]. Laser instruments designed specifically for dentistry have been available for about 30 years [10], and various protocols and parameters for periodontal applications have been developed [11]. For the purposes of this manuscript, the term ‘laser instruments’ or ‘laser’ is defined as those devices whose indications for use include debridement of the soft tissue side of the pocket. Thus a laser whose only purpose is for photoactivated disinfection and/or photobiomodulation will not be included in this review. While there have been numerous studies showing an additional benefit for laser use, there have been some papers describing no benefit. The purpose of this manuscript was to analyze manuscripts published between January 2010 and April 2020 that feature laser use adjunctive to periodontal debridement in randomized clinical trials. This review will answer the question: can adjunctive use of lasers provide an additional benefit during initial non-surgical therapy for chronic periodontitis when compared to a control group where a laser was not used at a 6-month evaluation?

## 2. Materials and Methods

### 2.1. Search Strategy

An electronic search was conducted between 15 April and 25 April 2020 relating to laser use as an adjunct in non-surgical periodontal therapy. Databases used were PubMed and the Cochrane Library with the following MeSH terms, keywords and their combinations:

(Laser OR diode OR Nd:YAG OR Er:YAG OR Er,Cr:YSGG OR CO_2_) AND (periodontitis OR periodontal) NOT (aPDT OR photodynamic OR PAD OR PDT OR photobiomodulation OR low level OR peri-implantitis OR endodontic OR orthodontic).

After applying the additional filters (published within the last 10 years, clinical trials in humans, and only English language reports), the preliminary number of 1294 articles was further reduced to 61.

Titles and abstracts of the above articles were independently screened by four reviewers via application of the following criteria. In case of any disagreements arising, these were satisfactorily resolved by discussions.

Inclusion criteria:

- Randomized controlled clinical trials;
- At least 10 patients per group;
- Chronic periodontitis;
- Laser used in test group;
- Interventions: the test groups received laser therapy additional to conventional treatment and one of the control groups received conventional treatment only;
- Follow up: at least 6 months.

Exclusion criteria:

- Case series/case reports/pilot studies;
- Studies without control group;
- Laser used as monotherapy in the test group;
- Surgical approach;
- Follow-up less than 6 months;
- Less than 10 patients per group;
- aPDT or other adjuncts applied.

**Figure 1:**
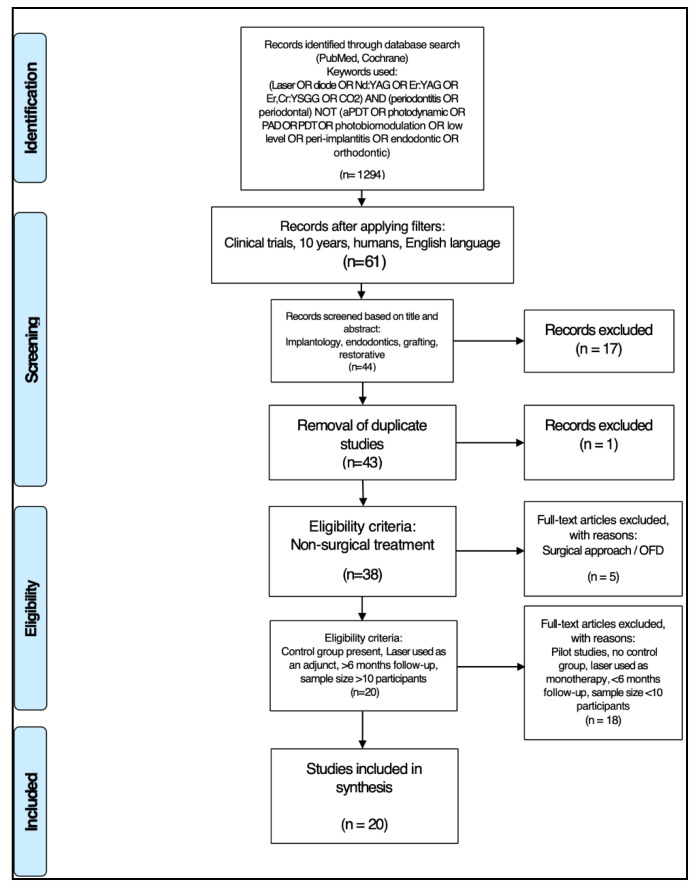
PRISMA flow-chart of selection criteria to determine article inclusion.

After screening and implementation of the eligibility criteria, a total of 20 articles were retained. In accordance with the PRISMA statement [12], details of the selection criteria are presented in Figure 1.

### 2.2. Data Extraction

Having reached a consensus regarding the selection of included articles, the four reviewers involved subsequently extracted data regarding:

- Citation (first author and publication year);
- Type of study/number of samples/pocket depth;
- Test/control groups;
- Examined parameters;
- Laser protocol/number of sessions involved;
- Follow-up;
- Outcome.

### 2.3. Quality Assessment

Following data extraction, articles were further scrutinized by evaluating their risk of bias. The Cochrane Risk of Bias tool [13] was modified according to the requirements of this systematic review.

The risk of bias was determined according to the number of “yes” or “no” responses to the parameters presented below, which were allocated to each study:

- Randomization?
- Sample size calculation and required sample numbers included?
- Baseline situation similar to that of the test group?
- Blinding?
- Parameters of laser use described appropriately, and associated calculations correct?
- Power meter used?
- Numerical results available (statistics)?
- No missing outcome data?
- All samples/patients completed the follow-up evaluation?
- Correct interpretation of data acquired?

The risk of bias is determined by the total number of “yes” answers to the above parameters, and hence classified as follows:

High risk = 0–4 ‘yes’ answers

Moderate risk = 5–7 ‘yes’ answers

Low risk = 8–10 ‘yes’ answers

## 3. Results

### 3.1. Primary Outcome

The primary goal of this systematic review was to critically appraise the treatment outcome of the adjunctive use of lasers in non-surgical periodontal therapy.

### 3.2. Data Presentation

The evaluation of the included studies is presented in Table 1.

### 3.3. Quality Assessment Presentation

A risk of bias assessment of studies included in this review is presented in Table 2.

In total 11/20 (55%) articles showed a low risk of bias with the following grading:

- 10/10 one article [24]
- 9/10 three articles [17,21,22]
- 8/10 seven articles [14,15,16,18,23,31,33]

Respectively, 9/20 (45%) articles showed a medium risk of bias with the following grading:

- 7/10 seven articles [19,25,26,27,28,29,30]
- 6/10 two articles [20,32]

Besides the sufficient description of the laser protocol used, the most common negative answers concerned (a) the power meter used and (b) the sample size calculation and required number included.

### 3.4. Analysis of Data

Regarding the primary outcome, positive outcomes in all examined parameters have been described in 3/20 (15%) studies [14,17,30], whereas positive results in some of the parameters and non-significant difference in the rest of them were shown in 11/20 (55%) [15,16,19,20,21,22,23,28,29,31,33].

No significant differences were found between the test and control groups in 4/20 (20%) studies [18,24,25,32].

Worse results in some of the examined parameters and no significant differences in the rest were shown in 2/20 (10%) [26,27].

Concerning the laser protocol applied, 14/20 (70%) showed incomplete laser parameter description [15,16,18,19,20,25,26,27,28,29,30,31,32,33]. The deficiencies concerned the following parameters:

- Tip or spot size: 5/14
- Frequency: 2/14
- Fluence/irradiance (either missing or wrongly calculated): 5/14
- Pulse duration: 9/14
- Irradiation time: 8/14

Power and energy per pulse were the only parameters, which were either indicated or could be calculated in all articles.

## 4. Discussion

### 4.1. Background

The purpose of this manuscript was to analyze, through a systematic review, manuscripts published between 2010 and 2020 that featured laser use adjunctive to periodontal debridement in randomized clinical trials. For purposes of this manuscript, ‘adjunctive’ was defined when scaling and root planning (SRP) using either ultrasonic driven scalers and/or hand scaling instruments was performed together other treatments such as a laser. Other adjunctive modalities have been developed, such as antimicrobial photodynamic therapy [34] or systemic and locally delivered anti-microbial medications [35] but will not be discussed here.

The term “non-surgical therapy” is defined as a protocol to remove as much calculus as possible to disrupt or eliminate the biofilm and accompanying microbes, and to reduce inflammation contributing to periodontal and peri-implant disease as initial therapy [36]. For several years in the latter part of the 20th century, some non-surgical procedures could have been considered essentially surgical ones, such as gingival curettage and removal of large amounts of root cementum. These two therapies have slowly been de-emphasized [37]. For clarity, surgical periodontal therapy is performed with excisional and incisional instruments, periosteal elevators, chisels, files, and tissue forceps. The site is exposed with a flap, then closed with sutures, and covered with a dressing [38]. Currently, surgery for treatment of chronic periodontitis has been de-emphasized in favor of non-surgical treatment. Many periodontal surgical procedures now include soft tissue and bone grafting to cover exposed roots and augment osseous structures for implant placement [39].

The use of lasers in periodontology can be seen in three areas of treatment: removal of diseased pocket lining epithelium, microbiocidal effect of lasers on pocket organisms and, for certain instruments, the removal of calculus deposits and root surface detoxification. When integrated into a sound approach to pocket reduction, all current dental wavelengths in the photoablative mode have been advocated for the removal of diseased epithelium [40,41,42,43,44,45,46,47]. Additionally, it should be noted that due to the local effects of photothermal laser action, laser treatment should be capable of effectively targeting periodontal tissues infected by bacteria [48,49].

The studies included in this manuscript represent a majority of the dental laser wavelengths; the citations’ wavelengths are: 532, 808, 810, 940, 1064, 2780, and 2940 nm. However, those of carbon dioxide (9300–10,600 nm) for which no paper matching our search and inclusion criteria could be found.

Table 1 lists the data found in each included manuscript in this review. The criteria of blinded, adequately sized groups, randomization, and statistics/data were fulfilled. Three studies included addition treatment: Dilsiz, et al. [23] and Giannopoulou, et al. [27] who used antimicrobial photodynamic therapy in one group and Lopes, et al. [33] who used a laser as monotherapy. Neither of these methods were part of our inclusion criteria and those groups were not analyzed in this review.

Several other aspects of the data will be discussed.

### 4.2. Group Selection at Baseline

Four groups had a large difference of male and female members of their study group. Sanz-Sanchez, et al. [20] reported 12 males and 28 females; Dilsiz, et al. listed 10 males and 14 females; Euzebio-Alves, et al. [24] indicated 13 males and 23 females; and Giannopoulou, et al. described 23 males and 9 females.

While the baseline measurements of the clinical parameters that each study measured were similar, we assigned a high rate of bias in the baseline grouping category to them.

### 4.3. Laser Parameters

Each included manuscript listed laser operating parameters. An analysis of those reported parameters will be further explained and scrutinized.

#### 4.3.1. Power 

There was a wide variety of how laser parameters were reported. Power parameters were stated in sometimes missing or confusing notations. Ciurescu, et al. [14], and Giannopoulou, et al. indicated a diode laser power parameter without any pulse notations, so the reader has to assume an undescribed continuous wave mode in which case that notation would be both peak and average power. Ciurescu, et al. also utilized two different power settings for the three diode sessions without explanation for the difference.

For free-running pulsed lasers, Zhou, et al. [15], Celik, et al. [16], Sanz-Sanchez, et al. [20] and Rotundo, et al. [32] did not list any power parameters, leaving the reader to perform the calculations.

For gated diode lasers, Saglam, et al. [22], and Dilsiz, et al. indicated power values without specification if it was the peak or average power.

Slot, et al. [26] refers the reader to a previously published study to find any laser parameters.

Qadri, et al. [30] uses a water-cooled pulsed Nd:YAG laser (1064 nm,) whose power ‘…was automatically controlled by the device.’ Ciurescu, Abduljabbar, Ustun, Saglam, Dilsiz, and Euzebio-Alves and their colleagues gave the most complete parameter descriptions.

Average power is one useful measurement when comparing clinical outcomes, especially among clinicians whose laser instrument’s panel has a power setting display. The parameters of adjunctive laser use for periodontal therapy can be found in the operating manual of the device and should be below those needed for excisional surgery [36].

#### 4.3.2. Pulse Duration

Zhou, et al. [18], Sanz-Sanchez, et al. [20], Ustun, et al. [21], Eltas and Orbak [28], Kelbauskiene, et al. [31], Rotundo, et al. [32], and Lopes, et al. [33] did not mention any pulse duration parameter. Most gated or free running pulsed lasers give the clinician the opportunity to select the pulse duration or ‘on’ time for the emission, which can significantly affect the tissue temperature rise.

#### 4.3.3. Power Meter Measurement

Except for Euzebio Alves, et al., no other study measured the actual power emission from the fiber/tip. That manuscript described a mean energy (sic) loss of approximately 20%.

Clinical experience has shown both a loss of transmission of photonic energy with fiber use and variation of that transmission among new fibers. A study of urology fibers confirmed those differences by using a power meter to measure them [50]. It is accepted that fiber construction may be of differing quality or purity [51] and some literature sources do allude to possible power losses along delivery fibers in a range of 5% to 20% [52]. Additional data show that there is a wide variation in the range of transmission for different infrared fibers, and that the loss in most of those fibers is quite high compared with silica fibers [53].

Thus, the output power mentioned the manuscripts is likely to be inaccurate.

#### 4.3.4. Fiber/Tip Size

Most studies reported a numerical tip diameter. Some provided no or minimal detail and/or used the manufacturer’s product name. Zhou described ‘A chisel-shaped fiber tip of 17 mm of length; Celik, et al. indicated a quartz tip (VARIAN 600/14); Abduljabbar, et al. [17] neglected to list the fiber size; Dereci, et al. used a ‘RFPT 5–14 360° firing tip’; Sanz-Sanchez, et al. reported a ‘proprietary sapphire tip’, and Lopes, et al. utilized a special application tip (1.1 · 0.5 mm); Eltas and Orbak [28] did not include any fiber size; That lack of information does not allow any analysis of the energy/power density delivered to the tissue.

#### 4.3.5. Irradiation Time

The described irradiation times were varied.

Four studies listed time per pocket: Ustun, et al. 20 s, Dilsiz, et al. 30 s, Euzebio Alves, et al. 20 s and Lopes 30 s.

Nine studies indicated the time per tooth: Ciurescu, et al. 30–60 s for diode and 10–15 s for Er,Cr:YSGG, Abduljabbar, et al. 60–120 s, Magaz, et al. 60 s, Saglam, et al. 20 s, Zingale, et al. [25] 30–45 s, Giannopoulou, et al. 60 s, Eltas and Orbak [28,29] 120 s, and Qadri, et al. 60–120 s.

Slot indicated “no more than 60 s per site” with no clear description of that term.

Zhou, Celik, Dereci, Sanz-Sanchez, Kelbauskiene, and Rotundo and their colleagues did not indicate any time parameters.

The irradiation time of laser photonic energy greatly influences the amount of tissue temperature necessary to remove diseased tissue and reduce pathogens [36,54]. The range of time listed in the studies included may have had an influence on the results.

### 4.4. Treatment Protocols

The conventional SRP methods in the studies showed some differences.

During SRP, Sanz-Sanchez, Dilsiz, Euzebio Alves and their colleagues (for both control and test groups), and Giannopoulou et al. (only for test sites) used only ultrasonic instrumentation.

Abduljabbar, Magaz, Dilsiz, Zingale, and Lopes and their colleagues utilized only hand instruments, e.g., curettes.

The remaining authors employed both ultrasonic and hand instruments: Ciurescu, Zhou, Celik, Dereci, Ustun, Salgam, Euzebio Alves (only for test teeth), Slot, Qadri, Kelbauskiene and Rotundo, along with their colleagues, as well as Eltas and Orbak [28,29].

These differences may not be significant and usually depend on the clinician’s skill. However, hand instrumentation does offer an enhanced tactile sense when removing the calculus. Euzebio Alves’s addition of this hand instrumentation to only the test group may also have affected the outcomes.

In the following studies, SRP was performed before the adjunctive laser therapy in the same session: Abduljabbar, Magaz, Sanz-Sanchez, Saglam, Slot, Giannopoulou, Qadri, Kelbauskiene, and Lopes and their colleagues, along with Eltas and Orbak [28,29].

However, Ustun, et al. and Zingale, et al. used an 810 nm diode laser, while Dilsiz, et al. employed a 532 nm KTP device before SRP therapy was performed.

It is well known that pigmented tissue has a high absorption by these wavelengths [54]. Caution is advised when using these wavelengths that could interact with the dark colored calculus on the root surface and cause thermal damage. Zhou, et al. and Rotundo, et al. used an erbium laser before SRP in the same session, but that laser family can safely remove calculus [36].

Three studies limited the number of teeth in their groups. Euzebio Alves, et al. and Eltas and Orbak [29] performed full mouth SRP but only evaluated one pair of contralateral teeth or one tooth per group, respectively. Lopes, et al. only used 4 sites for the control group (SRP) and the test group (laser + SRP.) This individual tooth study design could have limited significance, since all the patients had generalized periodontitis.

Several manuscripts described therapies spanning more than one session. Ciurescu, et al. listed multiple laser uses: initial treatment with a diode, a diode and Er,Cr:YSGG utilization one week later, and two months later, therapy consisted of a diode laser on all sites and an Er,Cr:YSGG on periodontal pocket depth (PD) ≥4 mm. Celik, et al. completed SRP and laser within 24 h. Dereci, et al. used the laser 3 times over 7 days. Sanz-Sanchez, et al. initiated laser treatment one week after SRP. Euzebio Alves, et al. performed diode therapy on the day after SRP and again at 7 days. Kelbauskiene and colleague’s therapy was an initial appointment of SRP + laser, followed by an average of two laser appointments, with a week between each visit.

The multiple laser treatments would certainly seem to provide additional benefit, although the results were comparable to single visit therapies.

### 4.5. Other Clinical Measurements and Influencing Factors

#### 4.5.1. Gingival Crevicular Fluid Level (GCF) Sampling

Abduljabbar, Ustun, Euzebio Alves (colony forming units (CFU) count), Giannopoulou, Qadri and Lopes and their colleagues as well as Eltas and Orbak [28,29]. noted split-mouth studies with GCF sampling; whereas Saglam, et al. used a parallel group design. Although both methods are acceptable, a question could be raised about the accuracy of split-mouth assaying since the pathogen population could have some similarities on both sides of the mouth.

It is worth noting that subject numbers should be sufficiently high to enable robust statistical analysis [55]. In the aforementioned GCF studies, the group sizes ranged from 19–52.

Moreover, two manuscripts only analyzed either one pair of contralateral teeth per patient (Euzebio Alves, et al.), or one tooth per patient (Eltas and Orbak) [29]. Considering the diversity of the flora in periodontal pockets, the addition of microbiologic analysis to conventional protocols should increase the quantification of the effectiveness of dental therapies [56].

#### 4.5.2. Smoking

While smoking has long been reported as one of the etiologic factors in periodontitis [57], Eltas and Orbak [28] reported that the smoker control and test group did not benefit from adjunctive laser use.

#### 4.5.3. Halitosis

Dereci’s adjunctive laser treatment showed significant reduction of halitosis, explained by the possible reduction of the volatile sulphur compounds (VSC) producing bacteria. This has some promising clinical application, since it has been reported that oral malodor is a common human attribute and can be an important reason to seek dental care [58].

#### 4.5.4. Follow-Up

A 6-month follow up minimum period was chosen as an inclusion criterion so that evaluation of the clinical outcomes would have more clinical significance, and a few of the studies exceeded that time frame. Dental practitioners always emphasize periodic visits for their patients with susceptibility to periodontal disease to decrease the likelihood of progressive and episodic disease. The American Academy of Periodontology’s position paper on periodontal maintenance (PM) states continuing care therapeutic appointments should be performed at intervals of less than six months, with the ideal schedule of PM every three months [59].

### 4.6. General Comments

The periodontal pocket occupies a three-dimensional space with irregular anatomy [60]. For meaningful photonic energy interaction, a careful and complete description of the protocol is necessary [61]. Some authors gave detailed descriptions of the direction and location of the laser emission during the irradiation time, but others did not. There were some explanations of coronal to apical motion, ‘sweeping’ motion, along with angulations of the tip. Those narratives are even more important when an end-emitting fiber or tip is used [62,63]. For example, Ciurescu described how the laser tip was ‘… introduced subgingivally to the bottom of the pocket and the laser firing was performed during sight sinusoidal retracting movements… until the entire accessible … surface was contacted. ‘ Abduljabbar describes placing ‘… the fiber into the periodontal pocket almost parallel to the tooth and moving from mesial to distal directions continuously on the buccal and the lingual aspect of the tooth. The fiber was held in a constant motion in contact with the pocket epithelial lining almost parallel to the long axis of the root.’ In contrast, Giannopoulou simply states that ‘… subgingival irradiation was performed with the diode laser for 60 s without any further description of the technique. A few mentioned the need to follow the manufacturer’s instructions, but most did not. As a result, any missing information increases the difficulty of reproducing the study and its results.

Some of the studies included only analyzed single rooted teeth, while others treated an entire quadrant which included multi-rooted teeth. As noted, some authors’ design had only one tooth. Given the complexity and variations of root anatomy, some of these study results may be criticized as not universally applicable for treatment of periodontitis.

From all studies evaluated, only two [26,27] found a negative outcome of the tested laser groups in only some of the examined parameters compared to SRP alone (pain, bleeding swelling on the first day and a higher percentage of remaining pockets >4 mm). This fact supports lasers as a safe adjunct to non-surgical periodontal therapy.

Table 2’s risk of bias shows that 55% of the studies [14,15,16,17,18,21,22,23,24,31,33] included in this review demonstrated a low risk of bias, and the remaining 45% [19,20,25,26,27,28,29,30,32] showed a moderate risk, with two lowest scores of 6.

## 5. Conclusions

The multi-factorial etiology and multiple treatment options when delivering initial periodontal therapy can offer challenges in attempting to standardize and analyze published papers. This systematic review found that 70% of the included studies [14,15,16,17,19,20,21,22,23,28,29,30,31,33] reported significantly better outcomes in certain clinical parameters, but no improvement in others. The remaining 30% of the manuscripts [18,24,25,26,27] reported no significant difference in any of the measurements. With consideration of correct parametry, lasers have an adjunctive role in initial non-surgical periodontal therapy. We encourage additional publication of long-term randomized clinical trials to confirm these additional benefits.

## Figures and Tables

**Table 1:** The results of the search showing the primary author and citation; the type of study/number of samples/pocket depth; test/control groups; examined parameters; laser protocol/number of sessions; follow up period; and outcomes.

Citation [Ref]	Type of Study/Number of Samples/Pocket Depth	Test/Control Groups	Aim/Approach	Laser Protocol/Number of Sessions	Follow-Up	Outcomes Including Stated PD Reduction and CAL Gain with Statistical Significance
Ciurescu et al., 2019 [14]	Parallel group RCT 38 Pts. Chronic periodontitis 1 pocket per quadrant ≥5 mm	Test (19) 940 Diode, Er,Cr:YSGG + US Control (19) US + hand instruments	PPD, BOP, CAL PCR microbiological. analysis	(i) diode, 940 nm: 1.5 W (day 0)–2 W (day 7), non-initiated 300 μm tip, sinusoidal retracting movements, 30 s for mono-rooted–60 s for multirooted/3 sessions: day 0, 7, 60 (ii) Er,Cr:YSGG 2780 nm: AvP 1.5 W, 30 Hz, 50 μs pulse, 45 mj/pulse, 500 μm radial tip, 10 s/mm pocket for mono-rooted–15 s/mm pocket for multi-rooted/2 sessions: day 7, 60	2 m tx and 6 months	Test group significantly better in PD, CAL, BOP Pg, Td, Tf, Pi, Pm, Fn, En compared to control. At 6 months, compared to control, PD reduction 1.19 mm; CAL 0.98 mm *p* < 0.001 for both.
Zhou et al., 2019 [15]	Randomized, single-blinded, controlled trial/25 patients/Split mouth, chronic periodontitis one pocket per quad ≥4 mm and BOP	one quadrant SRP + Er:YAG/one quadrant SRP	PPD, CAL, BI, PI at baseline, 3 months, and 6 months	Er:YAG (2940 nm) Hard tissue: 100 mJ 15 Hz Chisel tip coronal to apical in slow parallel paths. Soft tissue: 50 mJ 30 Hz Conical tip 800 μm	3 months, and 6 months	Er:YAG + SRP: PD and CAL sig. difference between groups at 3 + 6 mo. Differences clinically small (0.11 mm PD 0.2 mm CAL at 6 mo) *p* < 0.03 for both
Celik et al., 2019 [16]	Parallel group RCT 38 pts. 4 teeth in each quadrant, had a at least 4 pockets with PD ≥5 mm	SRP + Er:YAG 19 patients/SRP 19 patients	PPD, CAL, PI, BOP Microbiological evaluation using PCR	Er:YAG 2940 nm 150 mJ 10 Hz water irrigation 600 µm tip Coronal to apical 15–20°	3 months, 6 months	Test group significantly better than control in CAL, PD. At 6 months, compared to control, PD reduction 0.3–0.8 mm; CAL 0.5–0.8 mm *p* < 0.05 No significant difference in Pg Tf Td (*Porphyromonas gingivalis, Tannerella forsythia Treponema denticola)*
Abduljabbar et al., 2017 [17]	Split-mouth RCT/28 male patients with PD ≥4 mm	SRP + Nd:YAG/SRP	PI, BOP and PPD and GCF IL-1β (interleukin 1-beta)and TNF-α tumor necrosis factor-alpha) levels	Nd:YAG 1064 nm. Av P 4 W/80 mJ pp/50 Hz. Pulse width 350 μs peak power 240 W; Irrad: 1430 W/cm^2^ 60–120 s/tooth. Total energy/tooth 240–480 J.	3 months, and 6 months	Test group significantly better in PI, BOP, PD and GCF IL-1β and TNF-α levels compared with SRP alone. At 6 months compared to control, PD reduction listed as 1.0 mm *p* < 0.01.
Magaz et al., 2016 [18]	Split mouth RCT/30 pts. PD ≥4 mm + BOP	SRP + Er,Cr:YSGG/ SRP	PI, BOP, PPD, GR, CAL	Er,Cr:YSGG 2780 nm Av P 1.0 W, 50 mJ, 20 Hz. Air 10%/Water 15%, 60 s/tooth, 5–15°, 600 µm tip	6 weeks and 6 months	No significant difference between test and control groups.
Dereci et al., 2016 [19]	Parallel group RCT 60 pts/ 2 teeth with PD ≥5 mm + Halitosis	SRP + Er,Cr:YSGG/ SRP	PI, PPD, CAL, BOP, Halitosis VSC	Er,Cr:YSGG 2780 nm Av P: 1.5 W 30 Hz/Air 11%/Water 20%. 140 μs pulse. 600 µm radial firing tip. 10° apical to coronal 3 sessions/day 0, 2, 7	1, 3, 6 months	Test group significantly better in BOP and halitosis reduction (VSC) compared to control
Sanz-Sánchez et al., 2015 [20]	Parallel-group RCT /37 patients/ ≥4 teeth per quadrant, one with PD ≥4.5 mm, BOP Chronic periodontitis	SRP ultrasonic + Er:YAG (17) patients)/SRP ultrasonic (20 patients)	PD, REC, CAL, BOP	Er:YAG 2940 nm 160 mJ 10 Hz Sapphire tip	3, 6, 12 Months	Test group achieved a significantly lower percentage of PD ≥4.5 mm (*p* = 0.004) No significant difference between the groups for mean PD reduction (*p* = 0.08) or other clinical parameters. At 12 months, compared to control, PD reduction 0.16 and CAL 0.13 mm.
Üstün et al., 2014 [21]	Split-mouth RCT 19 pts PD 4–7 mm incisors or canines in two quadrants	SRP + diode laser 810 nm/ SRP	PI, GI, CAL, PPD GCF IL-1β flow cytometry	Diode 810 nm P 2.5 W Duty cycle 50% Av.P. 1.5 W 320 μm fiber, sweeping motion, slightly initiated tip, apical to coronal sweeping motion, 20 s per site/4 sites 1 session day 0	1, 3, and 6 months	Test group: At 1 month PPD, GI and GCF IL-1β significantly better *p* < 0.05 At 3 months PPD, CAL, GI and GCF IL-1β significantly better *p* < 0.05 At 6 months PPD, CAL, and GCF IL-1β significantly better *p* < 0.05. At 6 months compared to control, PD reduction 0.24 mm and CAL 0.45 mm. Both *p* < 0.05
Saglam et al., 2014 [22]	Parallel-group, RCT 30 pts. 2 teeth/quadrant PD ≥5 mm	SRP + diode 940 nm/ SRP	PI, GI, BOP, PPD, CAL GCF assay IL-1β, IL-6 (interleukin-6), IL-8 (interleukin-8), MMP-1 (matrix metalloproteinase-1), MMP-8 (matrix metalloproteinaise-8, TIMP-1(tissue inhibitor matrix metalloproteinase-1)	Diode 940 nm Av.P.1.5 W. Pulse length 20 ms on /20 ms off 10 s/buccal 10 s/lingual 15 J/cm^2^ fluence 300 μm tip Sweeping motion apical to coronal 1 session day 0	1, 3, and 6 months	Test group significant better compared to control: At 1 mo PPD, GI BOP, MMP-8 At 3 months BOP, TIMP-1 At 6 months PI, GI, TIMP-1. At 6 months compared to control, PD reduction 1.0 mm and CAL 0.2 mm Both *p* < 0.05.
Dilsiz et al., 2013 [23]	Split-mouth RCT/24 patients ≥4 non-adjacent teeth with PD ≥5 mm, BOP and bone loss Chronic periodontitis	SRP + KTP (potassium titanyl phosphate) (1)/ SRP + aPDT (2), SRP (3) (aPDT: MB + 808 nm) note: the aPDT group (2) was not included in our review.	PI, GI, BOP, PD, CAL	KTP 532 nm 2 applications: 0.8 W/50 ms on/50 off. 30 s. 200 μm/11.7 J/cm^2^ sweeping motion horizontally and coronally. 1 session: day 0	6 months	SRP + KTP group: Significant difference in PD and CAL compared to both other control groups. At 6 months in group 1 compared to control, PD reduction 2.08 and CAL 2,42 mm. Both *p* <0.001
Euzebio Alves et al., 2013 [24]	Split-mouth RCT/36 patients, one pair of contra-lateral single rooted teeth with PD >5 mm Chronic periodontitis	SRP + diode 808 nm/ SRP Full mouth debridement Only 36/36 teeth evaluated	CAL, PD, PI, BOP Microbiological Analysis CFU count Pg, Pi, Aa (*Porphyromonas gingivalis, Prevotella intermedia and* *Aggregatibacter actinomycetemcomitans)*	Diode 808 nm, 400 m fiber, 1.5 W CW, Irradiance 1193.7 W/cm^2^ Sweeping motion coronally parallel to tooth, 20 s/pocket. 2 sessions: day 0, 7	6 weeks, 6 months	No significant differences between groups
Zingale et al., 2012 [25]	Split-mouth RCT/25 pts. At least 5 pockets with PD ≥5–9 mm	2 test groups: Laser + SRP(1) Laser + SRP + laser sealing(2) 3 control groups: SRP only(3) papillae reflection + SRP + flap closure(4) No treatment(5)	PPD, BOP, CAL	Diode 810 nm 0.8 W CW 400 µm fibre initiated, 30–45 s per tooth (same parameters for curettage and sealing)	3, 6 months	No significant differences between treatment groups
Slot et al., 2012 [26]	Split-mouth RCT, 30 pts, At least two sites per quadrant with PD ≥5 mm, attachment loss ≥2 mm, BOP and bone loss. Moderate to severe generalised periodontitis	SRP + Nd:YAG/ SRP	Post-op pain, bleeding, swelling evaluation PD, REC, BOP	From reference Slot 2011	1 day post-op pain, bleeding, swelling evaluation 6 months PD, REC, BOP	Pain, bleeding, swelling reported significantly worse in test group No significant difference between groups in PD, REC, BOP
Giannopoulou et al., 2012 [27]	Split-mouth three-arm parallel-design RCT 32 pts. Per quadrant PD ≥5 mm +/CAL loss ≥ 2 mm + BOP	SRP + Diode 810 nm 1 Quadrant (1) SRP + aPDT 1 Quadrant (2) SRP 1 Quadrant (3) Note SRP + aPDT group (2) not included in our review.	PPD, BOP, REC, GCF levels of 22 different biomarkers, cytokines, acute-phase proteins evaluated	Diode laser 810 nm 1 W 60 s per tooth.	2 weeks, 2, 6 months	No significant differences between groups at any time Remaining pockets >4 mm 25% in test group vs 9% in both other groups (*p* = 0.034)
Eltas et al., 2012 (smokers) [28]	Split-mouth –4 armed RCT/52 patients 2 teeth per quadrant with PD 4–6 and bone loss, Chronic periodontitis	SRP + Laser (52 teeth from 26 patients- smokers) SRP + Laser (52 teeth from 26 patients non-smokers) SRP (52 teeth from 26 patients smokers) SRP (52 teeth from 26 patients non-smokers)	PI, CAL, PD, GI, GCF (volume)	Nd:YAG 1064 nm. Av P 1 W 100 mJ, 10 Hz Apical-coronal sweeping motion 120 s/ tooth.	1, 6 months	Statistically significant PD, GI, CGF improvement between non-smoker test group and all other groups at 6 months. (*p* < 0.05) At 6 months, the non-smoker group had PD reduction of 0.5 compared to all other groups. Statistically significant GI, CGF improvement between non-smoker test group and both smoker groups at 6 months. (*p* < 0.05) No significant differences in any parameters between test and control in the smoker group.
Eltas et al., 2012 [29]	Split-mouth, RCT 20 pts/40 teeth PD ≥ 4–6 mm/CAL loss ≥2 mm+	Nd:YAG + SRP(1tooth/patient) SRP. (1tooth/patient) Full mouth debridement. Only 2 teeth evaluated	PI, GI, PPD, CAL. GCF IL-1β and MMP-8 levels	Nd:YAG 1064 nm. Av P 1 W 100 mJ 10 Hz Apical-coronal sweeping motion 120 s/tooth 200 µm fibre	3, 9 months	9 months test group significantly better results in PPD, CAL, GI, and GCF values. At 9 months compared to control, PD reduction 0.91 and CAL 1.17 mm. Both *p* < 0.001. IL-1β and MMP-8 no sig. diff.
Qadri et al., 2011 [30]	Split-mouth RCT 22 pts at least six pockets of 4–8 mm on each side of the mandible	SRP + Nd:YAG/SRP	PI, GI, PPD, and marginal bone loss (measured on dig. BW radiographs. GCF vol.	Nd:YAG 1064 nm Av P. 4 W, 80 mJ/pulse, 50 Hz 350 μs pulse w/a 9 20–30° angulation Tx 60 and 120 s, depending on accessibility.	Median follow-up time 20 months (range 12–39 months)	PI, GI, PPD, marginal bone loss and GCF-volume significantly improved compared to control group Pocket depth reduction compared to control 1.61 mm at 20 months *p* < 0.001
Kelbauskiene et al., 2011 [31]	Split-mouth RCT/ 30 patients PD 4–6 mm on at least one site of a single rooted tooth. At least two quadrants are included. Chronic periodontitis	SRP + Er,Cr:YSGG 8–9 teeth per patient 509 sites test 579 control and	PI, BOP, PD, REC, CAL	Er,Cr:YSGG 2780 nm 1 W Av P. 20 Hz/600 μm tip 10%w/a 5–15° from coronal to apical (until root surface “acid-etched” appearance) Inner epithelial lining to the depth of pocket removed, 5 mm of outer epithelium removed, root surface conditioning. 3 sessions/day 0,7,14. 2nd and 3rd visits the amount of inner epithelial lining removed 1 mm less than the previous session.	2,3,6,12 months	Test group: Statistically significant differences in BOP, PD, and CAL compared to control. (*p* < 0.001) No differences for PI, REC
Rotundo et al., 2010 [32]	Split-mouth 4-armed RCT 27 pts. At least 2 teeth per quad PD ≥4–9 mm + BOP	SRP + Er:YAG (1) SRP (2) Er:YAG (3) Only supragingival debridement (4) Each one quadrant	PPD, BOP, PI, REC, CAL. Full mouth plaque & bleeding scores in addition. VAS pain.	Er:YAG 2940 nm 150 mJ 10 Hz 500 µm tip Water Coronal to apical 20° angulation	6 months	No significant difference in CAL between groups No *p*-values for all parameters except VAS and CAL
Lopes et al., 2010 [33]	Split-mouth 4 armed RCT/ 19 patients 4 non-adjacent sites in different quadrants with BOP and PD 5–9 mm.	SRP + laser(1)/ laser only(2)/ SRP(3)/ no treatment(4) 76 sites, 42 single or double rooted teeth, 34 multirooted teeth. Note: Group 2 was not included in our review because of monotherapy.	PD, GR, CAL, PI, GI, BOP GCF Microbiol analysis PCR Aa, Pg, Pi, Tf, Pn	Er:YAG, 2940 nm 100 mJ/10 Hz/AP 1.0 W, 12.9 J/cm^2^ 1.1 × 0.5 mm tip 30 s per site Apico-coronal movement 30° angulation Total irradiation time 180–240 s for each patient.	1, 3, 6, 12 months	SRP + laser statistically sig bacterial reduction at 6 and 12 months (*p* < 0.05) No statistically sig diff for PB, CAL, GI, BOP and PD.

The abbreviations/acronyms seen in the text are defined thus: RCT: Randomized Clinical Trial; CPD: Chronic Periodontal Disease; SRP: Scaling and Root Planing; Pt: Patient; US: Ultrasonic Scaling used for SRP; HI: Hand Instruments used for SRP; aPDT: antimicrobial photodynamic therapy; PD or PPD: Periodontal Pocket Depth; CAL: Clinical Attachment Loss; PI: Plaque Index; REC gingival recession; BOP: Bleeding on Probing; GI: Gingival Index; GCF: Gingival Crevicular Fluid Level; CFU: Colony Forming Units; PCR: Polymerase Chain Reaction; VSC: Volatile Sulphur Compounds; mJ: Millijoule; Hz: Hertz; W: Watts; AvP: Average Power; PP: Peak Power; CW: Continuous Wave.

**Table 2:** The risk of bias table showing ‘yes’ or ‘no’ answers to each of the parameters for each included manuscript. Those parameters are listed in Section 2.3 above, and the totals are indicated in the last column.

Citation [Ref]	Randomization	Sample Size Calculation and Required Number Included	Baseline Situation Similar	Blinding	Parameters of laser Use Described and Calculations Correct	Power-Meter Used	Numerical Results Available (Stats)	No Missing Out-Come Data	All Samples/Patients Completed the Follow-Up	Correct Interpretation of Data	Total Score/10
**PERIO**											
**Ciurescu et al., 2019 [14]**	Yes	No	Yes	Yes	Yes	No	Yes	Yes	Yes	Yes	8
**Zhou, X et al., 2019 [15]**	Yes	Yes	Yes	Yes	No	No	Yes	Yes	Yes	Yes	8
**Celik et al., 2019 [16]**	Yes	Yes	Yes	Yes	No	No	Yes	Yes	Yes	Yes	8
**Abduljabbar et al., 2017 [17]**	Yes	Yes	Yes	Yes	Yes	No	Yes	Yes	Yes	Yes	9
**Magaz et al., 2016 [18]**	Yes	Yes	Yes	Yes	No	No	Yes	Yes	Yes	Yes	8
**Dereci et al., 2016 [19]**	Yes	No	Yes	Yes	No	No	Yes	Yes	Yes	Yes	7
**Sanz-Sánchez et al., 2015 [20]**	Yes	Yes	No	Yes	No	No	Yes	Yes	Yes	No	6
**Üstün et al., 2014 [21]**	Yes	Yes	Yes	Yes	Yes	No	Yes	Yes	No	Yes	8
**Saglam et al., 2014 [22]**	Yes	Yes	Yes	Yes	Yes	No	Yes	Yes	Yes	Yes	9
**Dilsiz et al., 2013 [23]**	Yes	No	Yes	Yes	Yes	No	Yes	Yes	Yes	Yes	8
**Euzebio Alves et al., 2013 [24]**	Yes	Yes	Yes	Yes	Yes	Yes	Yes	Yes	Yes	Yes	10
**Zingale et al., 2012 [25]**	Yes	No	Yes	Yes	No	No	Yes	Yes	Yes	Yes	7
**Slot et al., 2012 [26]**	Yes	No	Yes	Yes	No	No	Yes	Yes	Yes	Yes	7
**Giannopoulou et al., 2012 [27]**	Yes	Yes	Yes	Yes	No	No	Yes	Yes	No	Yes	7
**Eltas et al., 2012 (Smokers) [28]**	Yes	Yes	Yes	Yes	No	No	Yes	Yes	Yes	No	7
**Eltas S et al., 2012 [29]**	Yes	No	Yes	Yes	No	No	Yes	Yes	Yes	Yes	7
**Qadri et al., 2011 [30]**	Yes	No	Yes	Yes	No	No	Yes	Yes	Yes	Yes	7
**Kelbauskiene et al., 2011 [31]**	Yes	Yes	Yes	Yes	No	No	Yes	Yes	Yes	Yes	8
**Rotundo et al., 2010 [32]**	Yes	Yes	Yes	Yes	No	No	No	Yes	Yes	No	6
**Lopes et al., 2010 [33]**	Yes	Yes	Yes	Yes	No	No	Yes	Yes	Yes	Yes	8
